# Probiotics ameliorate alveolar bone loss by regulating gut microbiota

**DOI:** 10.1111/cpr.13075

**Published:** 2021-06-07

**Authors:** Leming Jia, Ye Tu, Xiaoyue Jia, Qian Du, Xin Zheng, Quan Yuan, Liwei Zheng, Xuedong Zhou, Xin Xu

**Affiliations:** ^1^ State Key Laboratory of Oral Diseases & National Clinical Research Center for Oral Diseases West China Hospital of Stomatology Sichuan University Chengdu China; ^2^ Department of Cariology and Endodontics West China Hospital of Stomatology Sichuan University Chengdu China; ^3^ Department of Pediatric Dentistry West China Hospital of Stomatology Sichuan University Chengdu China; ^4^ Department of Dental Implantology West China Hospital of Stomatology Sichuan University Chengdu China

**Keywords:** alveolar bones, probiotics, oestrogen deficiency, osteoimmunology, Th17 cell, Treg cell

## Abstract

**Objectives:**

Oestrogen deficiency is an aetiological factor of postmenopausal osteoporosis (PMO), which not only decreases bone density in vertebrae and long bone but also aggravates inflammatory alveolar bone loss. Recent evidence has suggested the critical role of gut microbiota in osteoimmunology and its influence on bone metabolisms. The present study aimed to evaluate the therapeutic effects of probiotics on alveolar bone loss under oestrogen‐deficient condition.

**Materials and Methods:**

Inflammatory alveolar bone loss was established in ovariectomized (OVX) rats, and rats were daily intragastrically administered with probiotics until sacrifice. Gut microbiota composition, intestinal permeability, systemic immune status and alveolar bone loss were assessed to reveal the underlying correlation between gut microbiota and bone metabolisms.

**Results:**

We found administration of probiotics significantly prevented inflammatory alveolar bone resorption in OVX rats. By enriching butyrate‐producing genera and enhancing gut butyrate production, probiotics improved intestinal barrier and decreased gut permeability in the OVX rats. Furthermore, the oestrogen deprivation‐induced inflammatory responses were suppressed in probiotics‐treated OVX rats, as reflected by reduced serum levels of inflammatory cytokines and a balanced distribution of CD4^+^IL‐17A^+^ Th17 cells and CD4^+^CD25^+^Foxp3^+^ Treg cells in the bone marrow.

**Conclusions:**

This study demonstrated that probiotics can effectively attenuate alveolar bone loss by modulating gut microbiota and further regulating osteoimmune response and thus represent a promising adjuvant in the treatment of alveolar bone loss under oestrogen deficiency.

## Graphical abstract

Probiotics can promote intestinal barrier function by rescuing ‐ gut microbiota dysbiosis in estrogen‐deficient rats, which further suppressed the systemic inflammatory status and restored bone marrow Th17/Treg balance and consequently ameliorated inflammatory alveolar bone loss caused by periodontitis and periapical periodontitis. BM, bone marrow; LPS, lipopolysaccharide.

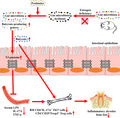

## 1 INTRODUCTION

Osteoporosis is a systematic bone disorder characterized by decreased bone mineral density (BMD) and compromised bone microarchitecture, leading to an increased risk of bone fracture.^1^ Postmenopausal osteoporosis (PMO) is the most common type of osteoporosis mainly caused by the cessation of ovarian function.^1^, ^2^ Oestrogen‐deficiency results in immune dysregulation, characterized by a skewed distribution and enhanced activity of Th17 cells (CD4^+^IL‐17A^+^cells).^3^ Th17 cells function as an osteoclastogenic subset of T‐helper cells that promote the release of inflammatory cytokines, such as RANKL, TNF‐α and IL‐17.^2^, ^3^ Elevated levels of circulating IL‐17 have been reported in either ovariectomized (OVX) mice or postmenopausal women.^4^, ^5^, ^6^ IL‐17 triggers osteoclastogenesis and simultaneously stimulates RANKL, TNF‐α and IL‐6 expression.^4^, ^7^, ^8^ Recent studies have demonstrated the close involvement of IL‐17 not only in the development of PMO but also in the progression of inflammatory arthritis and alveolar bone loss.^4^, ^9^, ^10^, ^11^

Periodontitis and periapical periodontitis are polymicrobial infectious diseases characterized by local inflammatory response within the supporting tissue of teeth, leading to alveolar bone loss.^12^, ^13^ Many studies have reported exacerbated alveolar bone resorption under oestrogen deficiency,^14^, ^15^, ^16^, ^17^ imposing challenges to the clinical treatment of these diseases. Conventional therapies of periodontitis and periapical periodontitis mainly rely on mechanical removal of plaque biofilm and root canal therapy. However, poor clinical treatment outcome has been noted in the elder women with PMO.^18^, ^19^, ^20^ The traditional PMO drugs such as bisphosphonates and RANK ligand inhibitor have been demonstrated effective in inhibiting osteoclast activity,^21^ but with non‐negligible adverse effects such as medication‐related osteonecrosis of the jaw (MRONJ),^22^, ^23^ limiting routine use of these drugs as an adjuvant treatment of periodontitis and periapical periodontitis.

Increasing evidence has indicated a close association between gut microbiota and bone metabolism, and the gut‐bone axis has been proposed.^24^, ^25^, ^26^ Aberrant gut microbiota is associated with decreased BMD and osteopenia.^27^, ^28^ In addition, the oestrogen‐depleted germ‐free mice present decreased expression of TNF‐α, RANKL and IL‐17 and increased BMD as compared to the SPF mice,^5^, ^29^ further underlining the critical roles of gut microbiota in skeletal homeostasis. Modulation of gut microbiota has been proposed as a potential approach to the management of skeletal disorders.^26^ Probiotics, which confer a health benefits on the host mainly via modulating gut microbiota, have demonstrated effectiveness in the treatment of inflammatory bowel disease (IBD), irritable bowel syndrome (IBS), antibiotic‐associated diarrhoea and necrotizing enterocolitis.^30^, ^31^ Recent studies have also demonstrated the protective effects of probiotics towards bone.^5^, ^32^, ^33^ Although specific mechanisms are still unclear, oral administration of certain probiotic strains has shown effectiveness in suppressing alveolar bone loss in animal models.^34^, ^35^, ^36^, ^37^ Our previous work showed that berberine, a natural alkaloid, was able to ameliorate periodontal bone loss by regulating gut microbiota of OVX rats,^38^ further underscoring the importance of gut microbiota modulation in the reversion of alveolar bone resorption. To further delineate the mechanism by which probiotics ameliorate alveolar bone loss, we hypothesize that probiotics can promote the intestinal barrier function, which subsequently alleviate osteoimmune response and consequently ameliorate periodontal and periapical bone loss under oestrogen deficiency. To validate this hypothesis, we administered probiotics to the OVX rats with either periodontitis or periapical periodontitis and investigated the effect of probiotics on the inflammatory alveolar bone loss. The effect of probiotics on gut barrier and osteoimmune response was further explored to delineate the underlying mechanisms.

## 2 MATERIALS AND METHODS

### 2.1 Animals and experimental design

A 10‐week‐old Sprague‐Dawley female rats (Da Shuo, China) were housed under specific pathogen‐free condition. After acclimatization for 1 week, rats were intraperitoneally anaesthetized by pentobarbital sodium (2%, 40 mg/kg) and bilaterally ovariectomized or subjected to sham surgery. At the same time, rats were administered with probiotics or vehicle until sacrificed. Rats in probiotics groups were supplemented with 1 × 10^7^ CFU/day commercially available infant probiotics production by intragastric gavage with the blunt syringe inserted into the stomach. The probiotics production contained 10 single strains including *Lactobacillus rhamnosu* HN001, *Bifidobacterium lactis* BI‐04, *Bifidobacterium animals* HN019, *Lactobacillus fermentium* SBS‐1, *Lactobacillus reuteri* 1e1, *Bifidobacterium longum* BB536, *Bifidobacterium breve* M16‐V, *Bifidobacterium infantis* Bi‐26, *Lactobacillus paracasei* Lpc‐37 (lifespace, Australia, later referred to as lsPro) or single probiotics strain of either *Bifidobacterium longu BL986* or *Lactobacillus* rhamnosus LRH09. Periodontitis or periapical periodontitis were induced three weeks after ovariectomy/sham operation. The periodontitis rat model was established according to the methods described by Li and Amar with minor modifications.^39^ Rats were intraperitoneally anaesthetized by pentobarbital sodium (2%, 40 mg/kg) and were ligated with a 5‐0 silk suture around the bilateral maxillary first molars to establish experimental periodontitis. *Porphyromonas gingivalis* ATCC 33277, which was anaerobically grown and resuspended to a concentration of 1 × 10^7^ CFU/mL in saline, was smeared on the silk suture every 3 days after ligation.^38^ The periapical periodontitis rat model was established as reported by Brasil with minor modifications.^17^ The tooth pulps of bilateral mandibular first molars were exposed to oral environment by making occlusal class I cavity near mesial margin using micro‐round bur in a high‐speed motor, leading to a spontaneous development of periapical periodontitis.

### 2.2 Specimens collection

Four weeks after ligation or pulp exposure, samples were harvested for analyses. Faeces were collected in 1.5‐mL sterile Eppendorf tubes and immediately stored at −80℃. Blood samples were collected from the abdominal aorta under intraperitoneal anaesthesia. Rats were then immediately sacrificed by cervical dislocation. 2‐cm segments of ileum from all rats were immediately excised and submerged into 1 mL of TriZol reagent for RNA isolation. Bilateral maxilla from rats representing periodontitis, bilateral mandibles from rats representing periapical periodontitis and 2‐cm segments ileum from all rats were removed and fixed in 4% paraformaldehyde for 24 hours. Femurs were dissected thoroughly free from soft tissue. The tips of the femurs were removed and bone marrow (BM) was harvested by inserting a syringe needle into one end of the bone and flushing with phosphate‐buffered saline (PBS).

### 2.3 Micro‐CT scanning and analysis

To evaluate the bone destruction and microarchitecture of alveolar bone and femur bone, micro‐CT was performed as previously described with minor modifications.^38^, ^40^, ^41^ Fixed bone specimens were placed in an airtight cylindrical sample holder and scanned with a micro‐CT (μCT50; SCANCO). The micro‐CT images were imported into CT‐Analyser software (version 1.13, Bruker, Kontich, Belgium) to qualitatively depict the alveolar bone loss and perform histomorphometric analysis of trabecular bone. As for alveolar bone, the scanning was performed at 70 kV and 200 mA with 300‐ms integration time. All samples were scanned in the sagittal position at a voxel resolution of 10 μm. As for rats with periodontitis, mesial and distal bone loss were quantified by measuring the average distance between the alveolar bone crest and the cemento‐enamel junction (CEJ) in sagittal images that contained complete mesial and distal root of maxillary first molar. 60 continuous sagittal images at maxillary first molar trifurcation, starting at the beginning of trabecular bone without the middle root, were selected for the analysis of trabecular bone at the region of interest (ROI). For each image, the ROI was a 5.2 mm^2^ rectangular region right below the top of bone septum between mesial and distal roots. The trabecular parameters of bone volume per tissue (BV/TV), trabecular number (Tb.N), trabecular separation (Tb. Sp) and trabecular bone pattern factor (Tb. Pf) at the ROI were measured and quantified. As for rats with periapical periodontitis, since the pulp exposure cavity was close to mesial margin of occlusal surface, 50 continuous sagittal images longitudinally through the centre section of mesial root of mandibular first molar were selected for the quantification of the volume of mandible resorption cavities at ROI. For each image, the ROI was a 5.2 mm^2^ rectangular region that contained the periapical resorption region of mesial root of mandibular first molar. Femurs were scanned at 70 kV and 200 mA with 300‐ms integration time in the transverse position at a voxel resolution of 12.5 μm.50 continuous images commencing 1200 μm above femoral condylar and extending horizontally for 750 μm were selected for trabecular bone analyses, and the trabecular parameters of BV/TV, Tb. N, Tb. Sp and Tb. Pf at the ROI were measured and quantified. The micro‐CT scanning and measurements were performed blindly by one experienced doctor.

### 2.4 Analysis of serum proinflammatory cytokine

Serum was isolated by centrifuging the blood after clotting at 1500 *g* for 10 minutes. The serum levels of TNF‐α and IL‐17A were assayed by ELISA kits (Invitrogen) according to the manufacturer's instructions.

### 2.5 Histologic analyses of alveolar bone and ileum tissue

Decalcified for 28 days, paraformaldehyde‐fixed and paraffin‐embedded 5‐μm thickness bone tissue sections were prepared for histologic analyses, which were performed with the method previously described with minor modification.^38^, ^41^ Sagittal sections of alveolar bone were subjected to observation after haematoxylin and eosin (H&E) staining, and those that included root furcation of maxillary first molar in periodontitis groups or mesial root of mandibular first molar in periapical periodontitis groups were selected to examine the periodontal or periapical destruction.

Osteoclasts in alveolar bone were examined by tartrate‐resistant acid phosphatase (TRAP) staining using the acid phosphatase leukocyte kit (Sigma, St. Louis, MO). OCN^+^ cells, Foxp3^+^ cells and IL‐17A^+^ cells in the alveolar bone were examined by immunohistochemistry (IHC) using specific primary antibodies (OCN, ab13420, Abcam; Foxp3, ab22510, Abcam; IL‐17A, ab136668, Abcam) and anti‐mouse HRP‐DAB cell & tissue staining kit (CTS002, R&D) or anti‐rabbit HRP‐DAB cell & tissue staining kit (CTS005, R&D systems). As for the quantification of positively stained cells in the alveolar bone of periodontitis, six high‐power fields (hpf) (×400) at ROI were randomly selected and the positively stained cells were enumerated by Image‐Pro Plus (IPP, Media Cybernetics, USA). Data were presented as the number of positively stained cells per square millimetre of bone marrow. As for the quantification of positively stained cells in the alveolar bone of periapical periodontitis, areas selected for cell enumeration were defined as those centred at a fixed distance from the mesial root apical foramen. Positively stained cells from 5 randomly selected areas at the periapical region were counted under hpf (×400) magnification, and data were presented as the number of positively stained cells per hpf.

Intestinal barrier integrity was also examined by H&E staining. Images captured at ×100 magnification were randomly selected, and morphologic features of intestinal villi including intestinal villus density (1/mm), intestinal villus height (mm), intestinal crypt depth (mm) and the ratio of villus height to crypt depth (V/C) were evaluated by IPP.

### 2.6 16S rRNA sequencing of gut microbiota

Genomic DNA was extracted and purified from faeces (3‐5 g) with the QIAamp DNA stool mini kit (QIAGEN). The resulting DNA was quantified with Quant‐iTTM PicoGreen reagent (Invitrogen). The sequencing of 16S rRNA amplicons (V1‐V3 region) was performed by MiSeq 300PE (Illumina MiSeq System) at Majorbio (Shanghai, China). Primers used in present study was 27F (5′‐AGAGTTTGATCCTGGCTCAG‐3′) and 533R (5′‐TTACCGCGGCTGCTGGCAC‐3′). A total of 151 465 sequence reads of faeces were generated from the amplicon library, with an average of 12 624 reads per sample. The sequences were clustered into 850 operational taxonomic units (OTU) in faeces at a similarity level of 97%. Bioinformatics were performed by Mothur and QIIME2.0 software, including quality control of raw data, taxonomic annotation based on the Silva database, taxonomy‐based comparisons at the OTU level, β‐diversity analysis including principal component analysis (PCA) and principal coordinates analysis (PCoA), dissimilarity analyses including analysis of similarity (ANOSIM) and non‐parametric multivariate analysis of variance (Adonis). The sequencing raw data were deposited in Sequence Read Archive (https://www.ncbi.nlm.nih.gov/Traces/sra; Accession nos. SRP285657).

### 2.7 Faecal butyrate quantification

Fresh faeces (10 mg per rat) were processed by the ether extraction method. A 20‐μL volume of the prepared sample solution was analysed by a high‐performance liquid chromatography (HPLC) system (model 1260; Agilent) with an HPLC column (4.0 mm × 250 mm, 5 μm, InertSustain C18; SHIMADUZ‐GL). The mobile phases were 0.2% H_3_PO_4_ solution (A phase) and methanol (B phase; Chromatographic Grade; Fisher Scientific), with a flow rate of 0.8 mL/min and a column temperature of 30℃. HPLC was performed with binary solvent‐delivery gradient elution with a detection wavelength of 210 nm.

### 2.8 RNA isolation and quantitative reverse transcription‐polymerase chain reaction (qRT‐PCR)

Intestinal RNA was isolated and purified from an ileum segment with TriZol Reagent (Invitrogen). Reverse transcription of RNA into cDNA was performed with the Primer‐Script RT Reagent Kit with gDNA Eraser (RR047A; Takara Bio). The expression of genes encoding intestinal tight junction (TJ) proteins, including occludin, claudin1, claudin 3, zo‐1 and Jam3 (encoding genes were named as *ocln*, *cldn 1*, *cldn 3*, *TJP1* and *jam 3,* respectively) was quantified with the β‐actin as internal control. The primer sequences are presented in Table 1. Relative quantitative analysis was performed with the 2^−ΔΔCT^ method.

**TABLE 1:** qRT‐PCR primer sequences for the TJ protein encoding genes

Primers	FP (5′ to 3′)	RP (5′ to 3′)
*ocln*	GCACGTTCGACCAATGCTCT	AGATGCCCGTTCCATAGGCT
*cldn 1*	ACTTTGCAGGCAACCAGAGC	TGCTGTGGCCACTAATGTCG
*cldn 3*	GGCCAGATGCAGTGCAAGAT	CCGAAGGCTGCCAGTAGGAT
*TJP1*	TCCCACAAGGAGCCATTCCT	GTCACAGTGTGGCAAGCGT
*Jam 3*	ACGGTCAGACTCAGCCATCT	AGAGTGCCTGTCTCCGAGTT
*β‐actin*	CTGAGCTGCGTTTTACACCCT	CGCCTTCACCGTTCCAGTTT

Abbreviations: TJ: tight junction.

Faecal RNA was isolated and purified with stool RNA kit (R6828‐01; OMEGA bio‐tec). Reverse transcription of RNA into cDNA was performed with the Primer‐Script RT Reagent Kit with gDNA Eraser (RR047A; Takara Bio). The expression levels of butyryl‐CoA:acetate CoA transferase (*But*) and butyrate kinase (*Buk*) of the gut microbiota were measured with the 16S rRNA gene as internal control. The primer sequences are presented in Table 2. Relative quantitative analysis was performed with the 2^−ΔΔCT^ method. As for the measurement of butyrate‐producing genera, faecal DNA was isolated and purified with stool DNA kit (QIAamp DNA stool mini kit, QIAGEN). The relative abundance of *Clostridium leptum* subgroup, *Clostridium coccoides* subgroup, *Fecalibacterium prausnitzii* and *Roseburia/E rectale cluster* was measured with the 16s‐univ‐1 gene as internal control. The primer sequences are presented in Table 3. Relative quantitative analysis was performed with the 2^−ΔΔCT^ method.

**TABLE 2:** qRT‐PCR primer sequences for butyryl‐CoA:acetate CoA transferase and butyrate kinase encoding genes

Primers	FP (5′ to 3′)	RP (5′ to 3′)
*But*	GCIGAICATTTCACITGGAAYWSITGGCAYATG	CCTGCCTTTGCAATRTCIACRAANGC
*Buk*	GTATAGATTACTIRYIATHAAYCCNGG	CAAGCTCRTCIACIACIACNGGRTCNAC

Abbreviations: Buk, butyrate kinase; But, butyryl‐CoA:acetate CoA transferase.

**TABLE 3:** qRT‐PCR primer sequences for the butyrate‐producing genera

Primers	FP (5′ to 3′)	RP (5′ to 3′)
*Clostridium leptum* subgroup	CGTCATCCCCACCTTCCTCC	GCAAGACAGTTTCAAGCGCA
*Clostridium coccoides* subgroup	AATGCCGCGGTGAATACGTT	GCACCTTCCGATACGGCTAC
*Fecalibacterium prausnitzii*	GATGGCCTCGCGTCCGATTAG	CCGAAGACCTTCTTCCTC
*Roseburia/E rectale* cluster	CKGCAAGTCTGATGTGAAAG	GCGGGTCCCCGTCAATTCC
SFB	TGAAGCGGAGGTAGATGGA	GCAACTATATAGCTGTATGCGG
*16s‐univ‐1*	GTGSTGCAYGGYTGTCGTCA	ACGTCRTCCMCACCTTCCTC

### 2.9 Intestinal permeability

After fasting and deprivation of water overnight, rats were gavage fed with 100 mg/mL of fluorescein isothiocyanate‐conjugated dextran (FITC‐dextran; 4.4 kDa, catalogue FD4, Sigma‐Aldrich) at 44 mg/100 g of body weight. 4 hours later, serum was collected from the abdominal aorta under intraperitoneal anaesthesia. The concentration of FITC‐dextran in serum was analysed by spectrophotofluorometry with excitation at 485 nm and emission at 528 nm, with reference to a standard of serially diluted FITC‐dextran (0, 150, 300, 600, 800, 1000 μg/mL). Serum lipopolysaccharide (LPS) levels were measured by an ELISA kit (Invitrogen).

### 2.10 Flow cytometry analysis of Th17/Treg cells

Fluorescence‐activated cell sorting (FACS) was used to evaluate the frequency (%) of Th17 cells (CD4^+^IL‐17A^+^ cells) and Treg cells (CD4^+^CD25^+^Foxp3^+^ cells) in the bone marrow of femur.

#### 2.10.1 Th17 cells

Bone marrow cells were incubated at 37℃ for 12 hours with DMEM cell high‐sugar medium mixed with 10% excellent fetal bovine serum and GolgiPulg (1 μg/mL) to block intracellular protein transport process. Single‐cell suspensions of bone marrow were then prepared in staining buffer, and 1 μL of specific FcR blocker was added to block the nonspecific staining mediated by fluorescent antibody FcR receptor. The cells were then stained with fluorescein isothiocyanate (FITC)‐labelled anti‐CD4 antibodies to detect surface markers. After membrane rupture, cells were intracellularly stained with phycoerythrin (PE)‐labelled anti‐IL‐17A antibodies. Cells were detected using a flow cytometer (Beckman, FC500, USA).

#### 2.10.2 Treg cells

Single‐cell suspensions of bone marrow were prepared in staining buffer. The cells were then stained with FITC‐labelled anti‐CD4 antibodies, followed by PE anti‐CD25 antibodies. After the fixation and membrane rupture, cells were stained with allophycocyanin (APC)‐labelled anti‐Foxp3 antibodies. Cells were detected using a flow cytometer (Beckman, FC500, USA).

### 2.11 Butyrate treatment

To further validate the role of intestinal butyrate in maintaining gut permeability and preventing skewed Th17/Treg‐induced bone resorption, additional OVX/sham rats were gavage‐fed with sodium butyrate (400 mg/kg) every day for 8 wk until sacrifice. Serum, ileum and bone marrow cells were collected for further analyses with the same methods as described above.

### 2.12 Statistical analysis

All data were statistically analysed by SPSS v25.0 (Statistical Product and Service Solutions, International Business Machine Inc, USA). Differences between groups were evaluated by one‐way ANOVA with Bonferroni correction for multiple comparisons or by the Kruskal‐Wallis H test with post hoc tests applying the Nemenyi test for multiple comparisons. All quantified data are presented as mean ± SD. 2‐tailed *P* < .05 was considered statistically significant, and *P* values were rounded up to the forth decimal.

## 3 RESULTS

### 3.1 Probiotics restore the gut permeability of OVX rats by enriching butyrate‐generating bacteria

**FIGURE 1:**
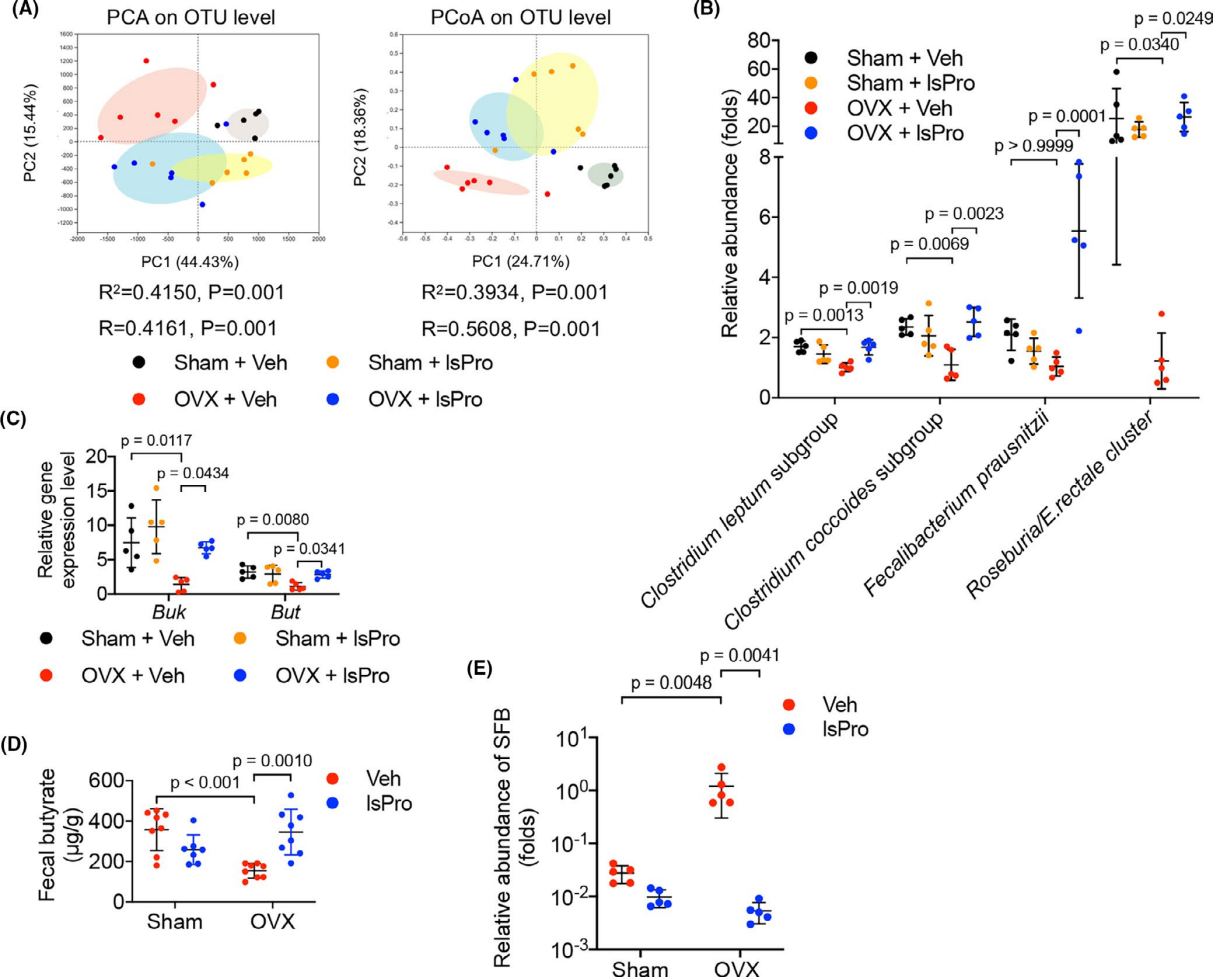
Probiotics enrich butyrate‐producing gut microbiota in OVX rats. A, The β‐diversity analyses (PCA and PCoA) of gut microbial community based on Bray‐Curtis distance. B, The abundance of butyrate‐producing genera relative to control (OVX + Veh) as quantified by qRT‐PCR. C, The relative expression levels of butyryl‐CoA:acetate CoA transferase (But) and butyrate kinase (Buk) of the gut microbiota as quantified by qRT‐PCR. D, Quantification of faecal butyrate concentration by HPLC. E, The abundance of SFB relative to control (OVX + Veh) as quantified by qRT‐PCR. Data are presented as the mean ± SD, n = 5‐8 rats per group. The p values are indicated above the horizontal bars. But, butyryl‐CoA:acetate CoA transferase; Buk, butyrate kinase; lsPro, life space probiotics; OTU, operational taxonomic unit; PCA, Principal components analysis; PCoA, principal coordinates analysis; Veh, Vehicle

Principal components analysis (PCA) and principal coordinate analysis (PCoA) based on Bray‐Curtis distance showed significant alteration of gut microbiota on operational taxonomic unit (OTU) level in OVX rats. The altered microbial community structure was reversed by the administration of probiotics (Figure 1A). More importantly, intragastric administration of probiotics significantly elevated the levels of butyrate‐producing genera that were deprived in OVX rats, including *Clostridium leptum* subgroup, *Clostridium coccoides* subgroup, *Fecalibacterium prausnitzii* and *Roseburia/E rectale cluste* (Figure 1B). Consistently, we found that transcripts encoding butyrate synthesis‐associated enzymes, including *But* and *Buk* were downregulated in the OVX rats and the administration of probiotics significantly upregulated *But* and *Buk* expression (Figure 1C). In parallel, the scarcity in butyrate as observed in the intestine of OVX rats was reversed by the intragastric administration of probiotics (Figure 1D). Furthermore, we observed an increased abundance of gut segmented filamentous bacteria (SFB) in OVX rats, and administration of probiotics significantly reduced the intestinal level of SFB (Figure 1E).

**FIGURE 2:**
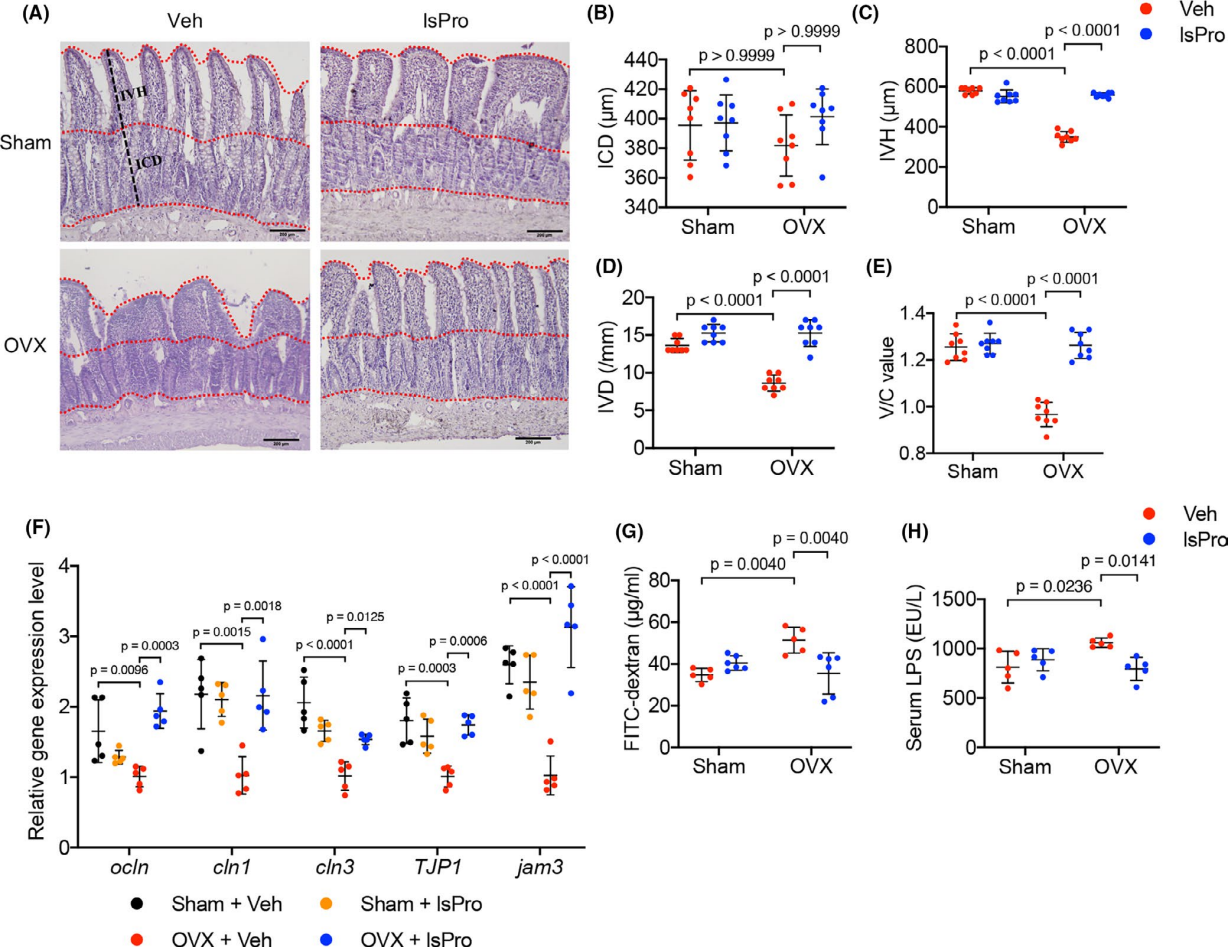
Probiotics restore the gut permeability of OVX rats. A, Representative images of H&E staining of the ileum mucosa (Scale bar = 200 μm). B‐E, Quantitative analyses of ICD, IVH, IVD and V/C value of the ileum mucosa, respectively. F, The relative expression levels of intestinal epithelial TJ proteins as quantified by qRT‐PCR. G, The serum level of FITC‐dextran as determined by spectrophotofluorometry. H, The serum level of LPS as determined by ELISA. Data are presented as the mean ± SD, n = 5‐8 rats per group. The p values are indicated above the horizontal bars. ICD, Intestinal crypt depth; IVD, Intestinal villus depth; IVH, Intestinal villus height; lsPro, life space probiotics; TJ, tight junction, Veh, Vehicle; V/C, villus height to crypt depth

Further morphologic analyses of the intestinal villi showed no significant difference in intestinal crypt depth (ICD) in the OVX rats as compared to the sham‐operated controls (Figure 2B). However, significant decrease in intestinal villus height (IVH), intestinal villus density (IVD) and V/C value was observed in OVX rats as compared to the sham‐operated rats (Figure 2C‐E), suggesting a compromised gut barrier (Figure 2A). More importantly, intragastric administration of probiotics effectively improved the intestinal barrier of the OVX rats via restoring the normal morphology of intestinal villi as reflected by IVH, IVD and V/C value (Figure 2A,C‐E).

The gut permeability of the OVX rats was further evaluated. qRT‐PCR data showed a decreased expression of TJ proteins, including Occludin, Claudin1, Claudin3, ZO1 and JAM3 (the encoding genes of which were *ocln*, *cldn 1*, *cldn 3*, *TJP1* and *jam 3* separately) in the intestinal epithelia of OVX rats and administration of probiotics significantly upregulated the expression of these TJ proteins‐related genes (Figure 2F). Serum levels of FITC‐dextran and LPS were further measured to evaluate the intestinal paracellular permeability. Consistently, elevated levels of serum FITC‐dextran and LPS were observed in OVX rats as compared to the sham‐operated controls (Figure 2G,H), suggesting an enhanced gut permeability under oestrogen deficiency. Intragastric administration of probiotics promoted the gut barrier in the OVX rats as reflected by the decreased leakage of FITC‐dextran and LPS (Figure 2G,H). In addition, gavage feeding of exogenous butyrate upregulated the expression of TJ proteins and decreased the intestinal leakage of FITC‐dextran in OVX rats (Figure S1A,B), further suggesting that butyrate is critical for gut permeability and probiotics protect the gut barrier of OVX rats likely by enriching the butyrate‐generating bacteria.

### 3.2 Probiotics alleviate bone resorption and normalize aberrant osteoimmune response in OVX rats

**FIGURE 3:**
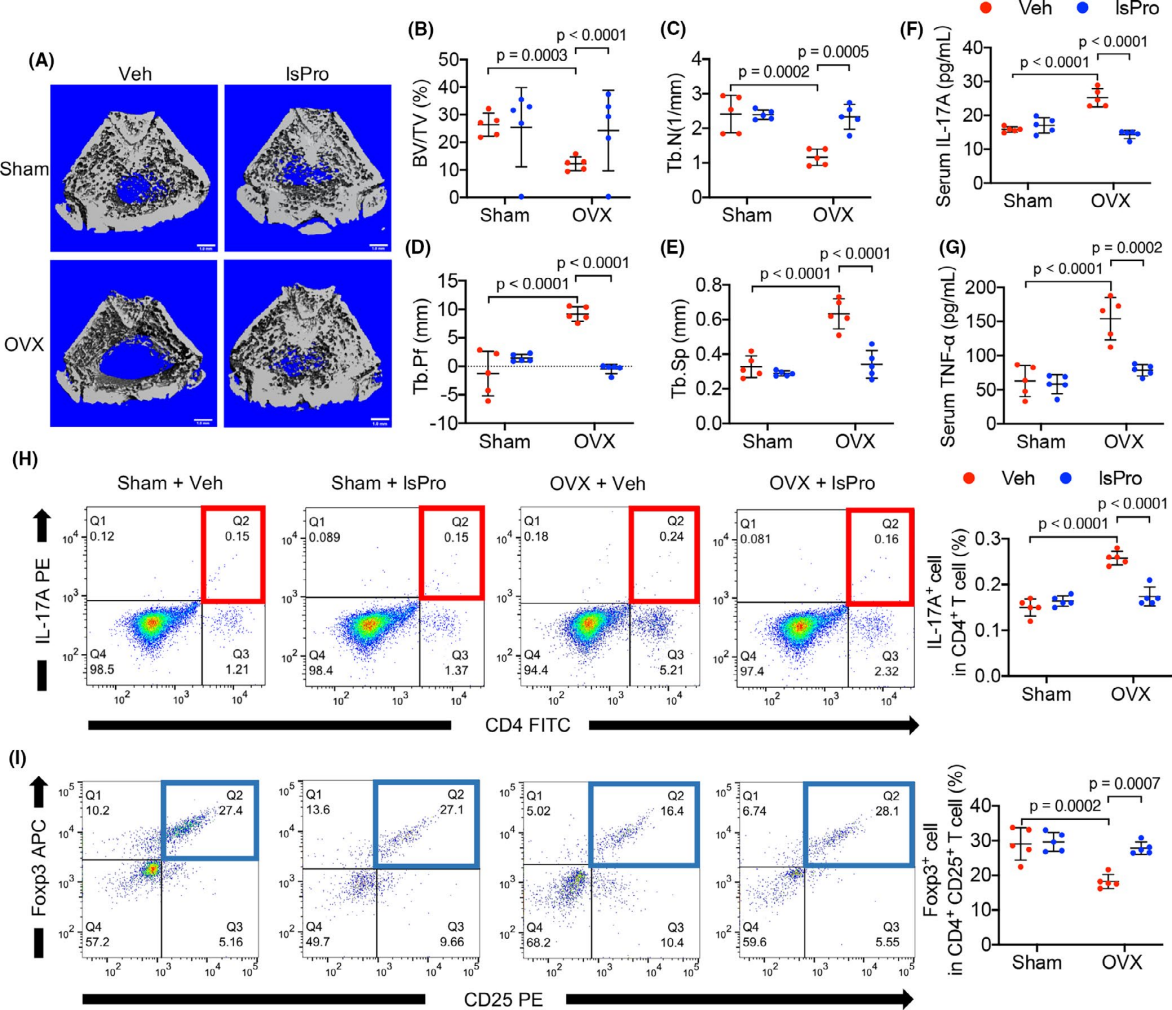
Probiotics ameliorate bone loss and rescue Th17/Treg imbalance in OVX rats. A, Micro‐CT reconstruction of femoral bone structure. B‐E, Quantitative analyses of BV/TV, Tb.N, Tb.Pf, Tb.Sp of the femoral bone by micro‐CT. F, Serum level of IL‐17‐A. G, Serum level of TNF‐α. H, Representative FACS plots of IL‐17A^+^ cells in the CD4^+^ T‐cell subset in the BM and quantitative analysis. I, Representative FACS plots of Foxp3^+^ cells in CD4^+^CD25^+^ T‐cell subset in the BM and quantitative analysis. Data are presented as the mean ± SD, n = 5 rats per group. The p values are indicated above the horizontal bars. BV/TV, bone volume per tissue volume; lsPro, life space probiotics; Tb.N, trabecular number; Tb.Pf, trabeculae pattern factor; Tb.Sp, trabecular separation; Veh, Vehicle

Micro‐CT analyses of the femoral cancellous bone of the OVX rats showed a typical osteoporosis phenotypes, as reflected by the decrease in BV/TV and Tb.N, while increase in Tb. Sp and Tb. Pf (Figure 3A‐E). Administration of probiotics significantly rescued the bone loss of OVX rats (Figure 3A‐E) and the complex probiotic showed better protective effects as compared to the single strain of *Bifidobacterium longum* BL986 and *Lactobacillus rhamnosus* LRH09 (Figure S2A‐E).

Analysis of serum inflammatory cytokines showed an increased level of IL‐17A and TNF‐α in the OVX rats as compared to the sham‐operated controls, and intragastric administration of probiotics countered the elevation of these inflammatory cytokines (Figure 3F,G). We further analysed the osteoimmune response with flow cytometry. A skewed distribution of CD4^+^IL‐17A^+^ Th17 cells and CD4^+^CD25^+^Foxp3^+^ Treg cells in the bone marrow of OVX rats was observed as reflected by a higher frequency of CD4^+^IL‐17A^+^ Th17 cells (Figure 3H) and lower frequency of CD4^+^CD25^+^Foxp3^+^ Treg cells (Figure 3I). Intragastric administration of probiotics reconstituted the Th17/Treg balance by decreasing CD4^+^IL‐17A^+^ Th17 cell ratio (Figure 3H) and increasing CD4^+^CD25^+^Foxp3^+^ Treg cell ratio from the BM, respectively (Figure 3I). In addition, exogenous butyrate treatment also rescued the Treg/Th17 imbalance (Figure S3A,B), further suggesting the linkage between gut permeability and abnormal osteoimmune response.

### 3.3 Probiotics ameliorate periodontal bone loss in OVX rats

**FIGURE 4:**
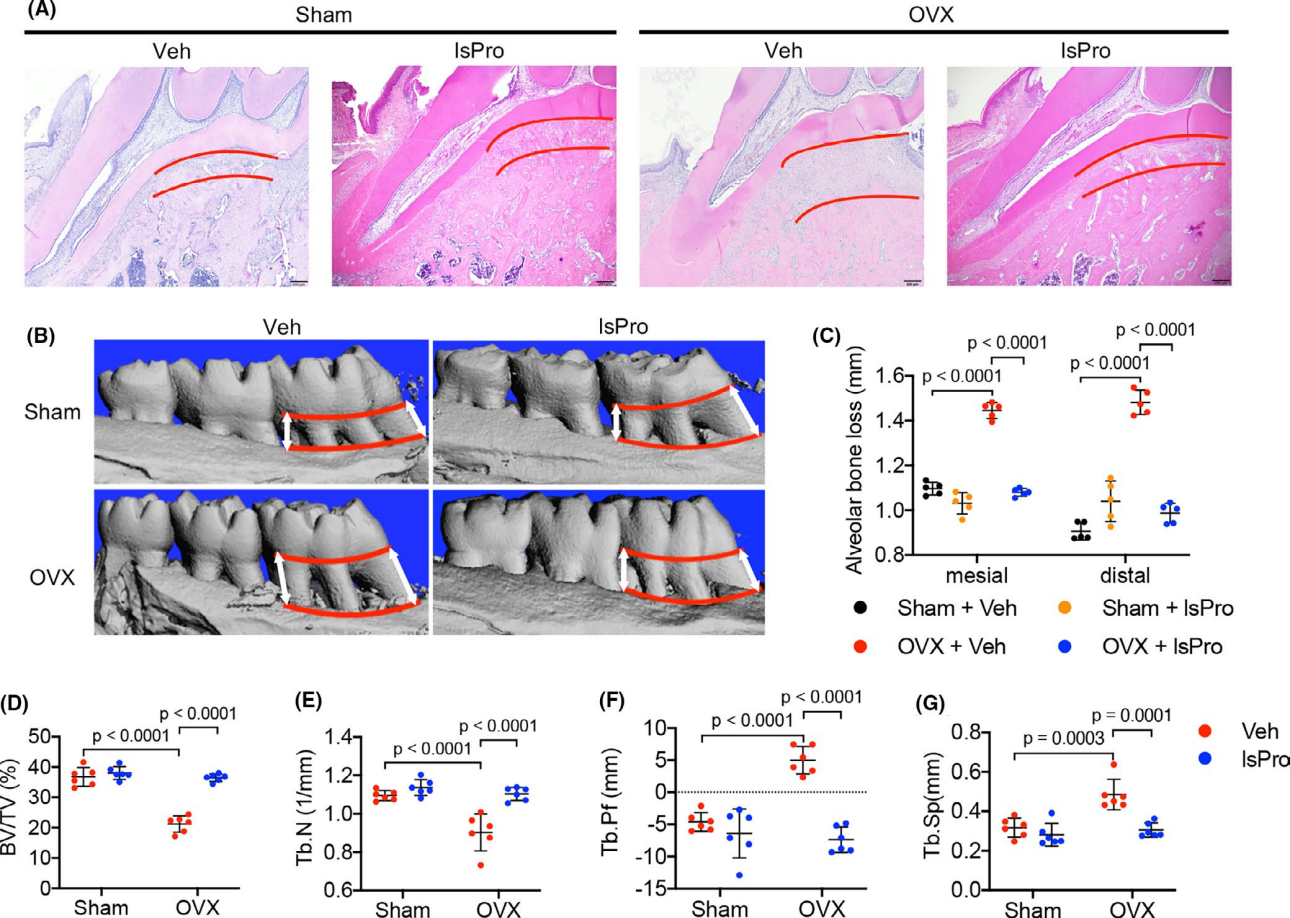
Probiotics ameliorate periodontal bone loss in OVX rats. A, H&E staining of the periodontal bone loss at root furcation of maxillary first molar (distance between the red lines). Scale bar = 200 μm. B, Representative images of micro‐CT reconstruction of periodontal bone loss between the mesial and distal sites (distance between the red lines) of maxillary first molars. C, Quantitative analyses of mesial and distal alveolar bone loss by micro‐CT. D‐G, Micro‐CT analyses of BV/TV, Tb.N, Tb.Pf and Tb.Sp of the alveolar bone, respectively. Data are presented as the mean ± SD, n = 5‐8 rats per group. The p values are indicated above the horizontal bars. BV/TV, bone volume per tissue volume; lsPro, life space probiotics; Tb.N, trabecular number; Tb.Pf, trabeculae pattern factor; Tb.Sp, trabecular separation; Veh, Vehicle

**FIGURE 5:**
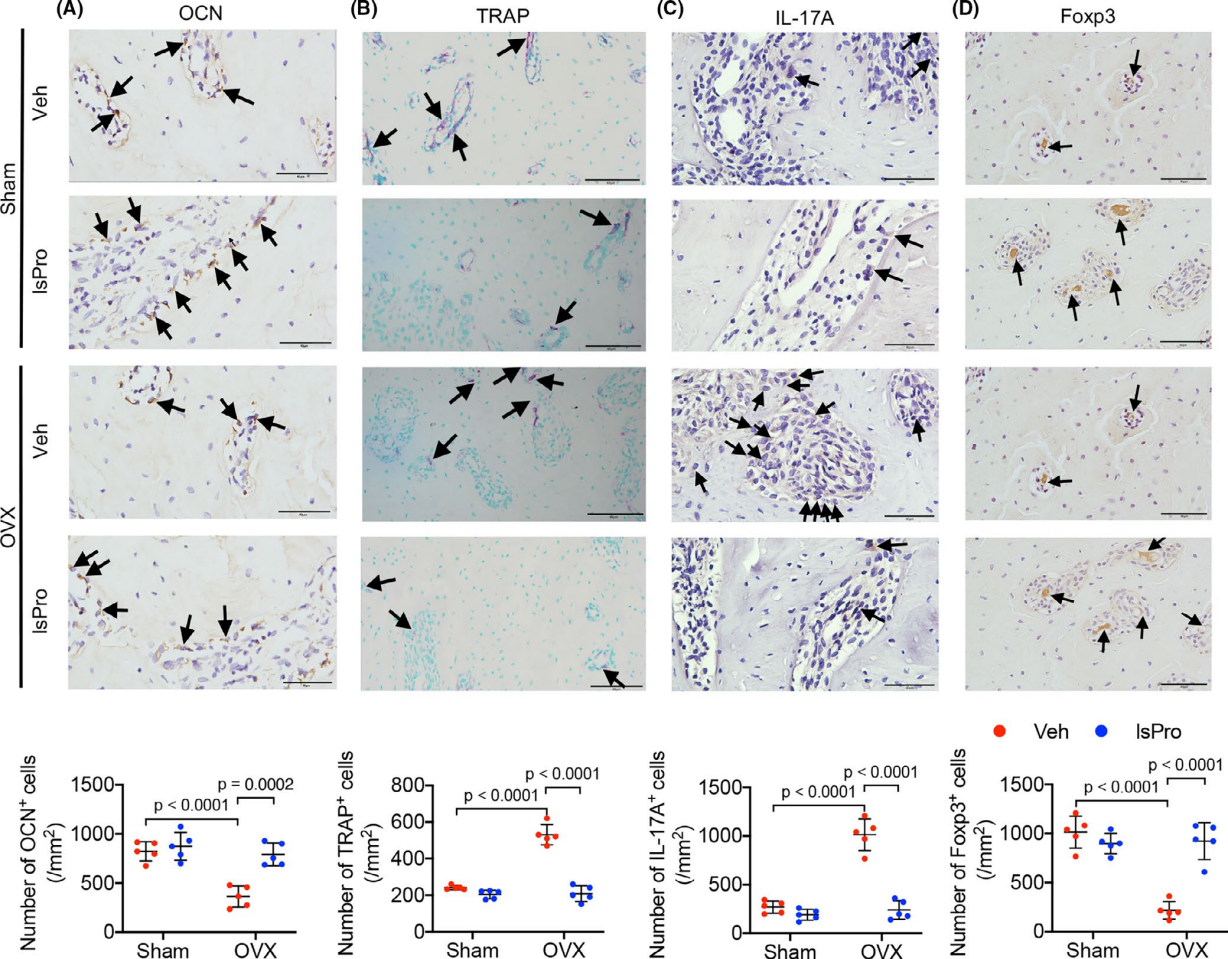
Probiotics attenuate periodontal bone resorption in OVX rats. A‐D, Representative images of OCN^+^ cells, TRAP^+^ cells, IL‐17A^+^ cells and Foxp3^+^ cells at the maxillary first molar and quantitative analyses of positive cells number per bone surface, respectively. Black arrows indicate positively stained cells. Scale bar = 40 μm. Data are presented as the mean ± SD, n = 5 rats per group. The p values are indicated above the horizontal bars. lsPro, life space probiotics; OCN, osteocalcin; TRAP, tartrate‐resistant acid phosphatase; Veh, Vehicle

The effects of oestrogen‐deficiency and probiotics treatment on the periodontal bone resorption were further investigated. Oestrogen deficiency exacerbated the alveolar bone resorption in periodontitis (Figure 4A‐C), resulting in decreased BV/TV and Tb.N, whereas increased Tb. Sp and Tb. Pf in the alveolar bone (Figure 4D‐G). Intragastric administration of probiotics significantly ameliorated periodontal bone loss of OVX rats (Figure 4A‐G) and the complexprobiotic showed better protective effects as compared to the single strain of *Bifidobacterium longum* BL986 and *Lactobacillus rhamnosus* LRH09 (Figure S4). Further histological analyses showed a reduced osteocalcin^+^ (OCN) osteoblasts and increased TRAP^+^ osteoclasts per bone surface in the OVX/periodontitis rats as compared to the sham/periodontitis controls. The decreased numbers of osteoblasts suggest decreased bone formation and the enhanced numbers of osteoclasts suggest active resorption (Figure 5A,B). Probiotics treatment significantly countered the elevated activity of bone resorption by increasing OCN^+^ cells and decreasing TRAP^+^ cells in the alveolar bone (Figure 5A,B). Furthermore, an increased number of IL‐17A^+^ cells and decreased Foxp3^+^ cells per bone surface were observed in OVX/periodontitis rats and this skewed CD4^+^ T cell‐mediated osteoimmune response was rescued by the administration of probiotics (Figure 5C,D).

### 3.4 Probiotics ameliorate periapical bone loss in OVX rats

**FIGURE 6:**
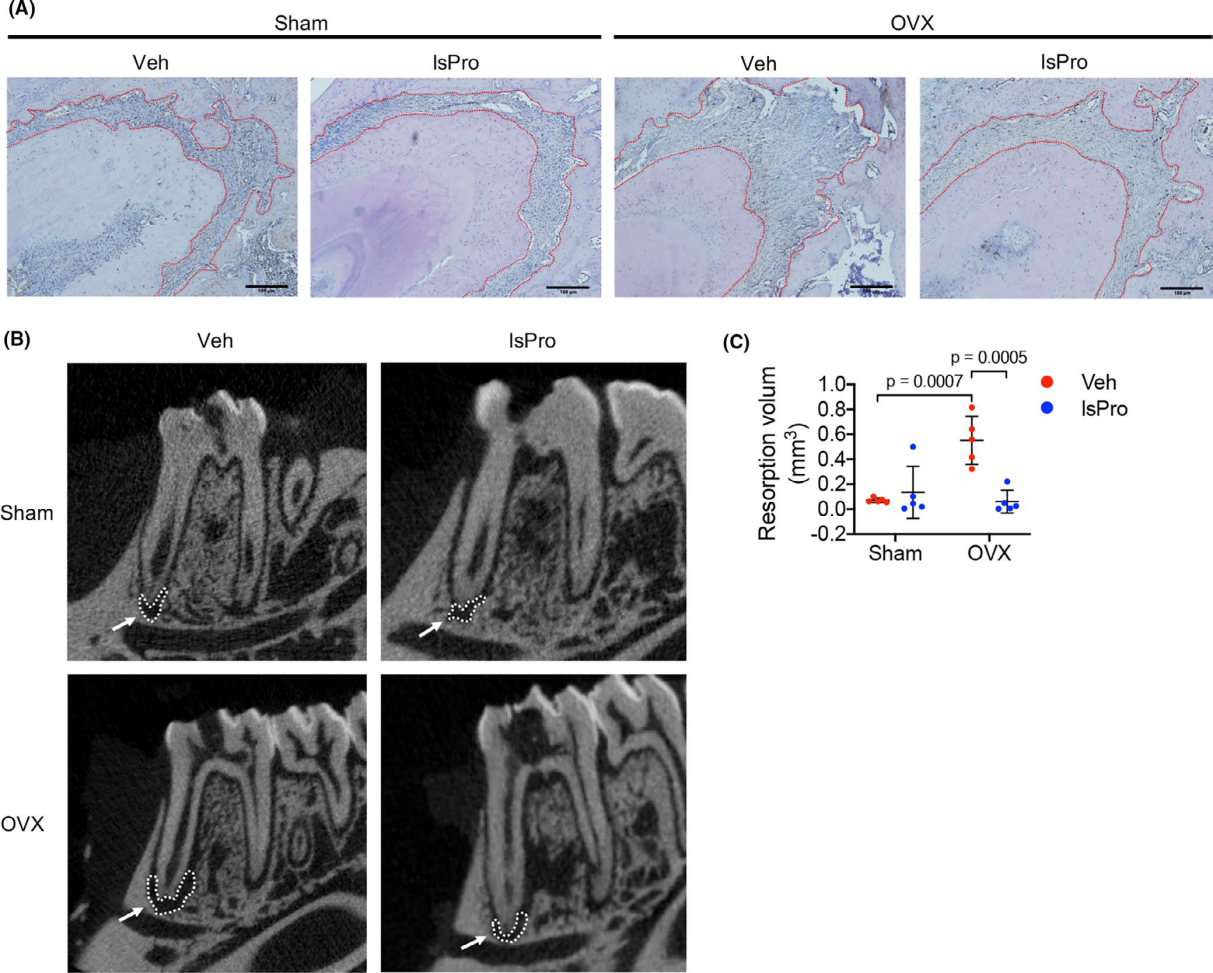
Probiotics ameliorate periapical bone loss in OVX rats. A, H&E staining indicating periapical bone loss of mesial root of mandibular first molar (area within the red line). Scale bar = 100 μm. (B,C) Representative micro‐CT images of periapical lesion of mesial root of mandibular first molar (white arrow) and quantitative analysis of resorption volume in periapical regions. Data are presented as the mean ± SD, n = 5‐8 rats per group. The p values are indicated above the horizontal bars. lsPro, life space probiotics; Veh, Vehicle

**FIGURE 7:**
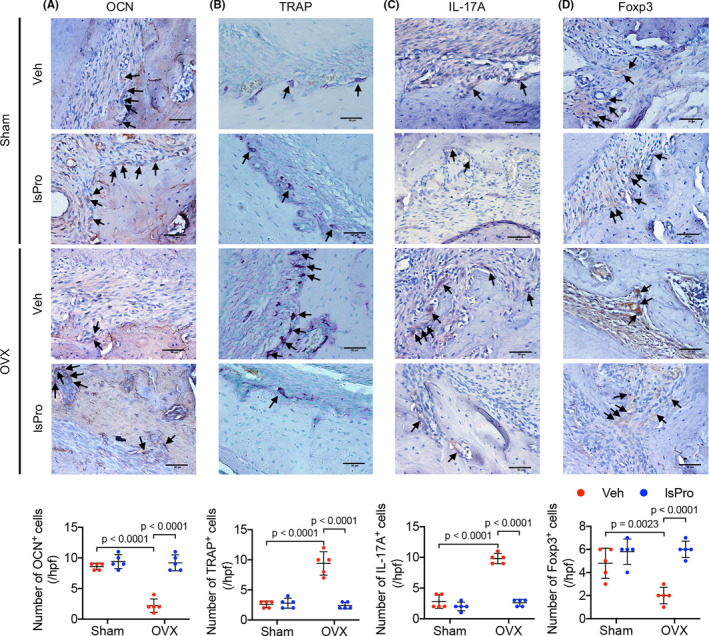
Probiotics attenuate periapical bone resorption in OVX rats. A‐D, Representative images of OCN^+^ cells, TRAP^+^ cells, IL‐17A^+^ cells and Foxp3^+^ cells at the periapical area of mesial root of mandibular first molar and quantitative analyses of positive cells number per hpf, respectively. Black arrows indicate positively stained cells. Scale bar = 50 μm. Data are presented as the mean ± SD, n = 5 rats per group. The p values are indicated above the horizontal bars. Hpf, high‐power fields; lsPro, life space probiotics; OCN, osteocalcin; TRAP, tartrate‐resistant acid phosphatase; Veh, Vehicle

The periapical periodontitis rat model was further established to investigate the effect of oestrogen deficiency and probiotics on the inflammatory alveolar bone loss. Oestrogen deficiency aggravated alveolar bone loss in periapical bone region (Figure 6A‐C), while intragastric gavage of probiotics effectively ameliorated the periapical bone loss of OVX rats (Figure 6A‐C). Further histological analyses showed a resorption‐dominant condition in the periapical bone region of OVX/periapical periodontitis rats, as reflected by decreased cell number of OCN^+^ osteoblasts and increased cell number of TRAP^+^ osteoclasts per high‐power field (hpf) (Figure 7A,B). Intragastric administration of probiotics significantly reversed the periapical bone resorption by elevating the number of OCN^+^ cells and reducing the number of TRAP^+^ cell per hpf (Figure 7A,B). Moreover, the skewed distribution of Th17/Treg cells in the periapical bone region of OVX/periapical periodontitis rats was also rescued by the treatment of probiotics, as reflected by decreased number of IL‐17A^+^ cells and increased number of Foxp3^+^ cells per hpf (Figure 7C,D).

## 4 DISCUSSION

Periodontitis and periapical periodontitis are common oral diseases that cause gradual loss of alveolar bone and consequently tooth loss. Poor oral hygiene and systemic diseases such as diabetes and postmenopausal osteoporosis predispose individuals to the development and progression of periodontitis. More importantly, diabetes and PMO can compromise the clinical outcome of conventional treatment such as oral prophylaxis and root canal therapy,^42^, ^43^, ^44^ imposing challenges on the clinical management of these diseases, particularly in the population of elder women. The current study demonstrated that probiotics can ameliorate the alveolar bone loss in oestrogen‐deficient rats via modulating the gut microbiota and thus represent a promising adjuvant to the treatment of periodontitis and periapical periodontitis.

Recent studies have revealed the critical role of Th17/Treg balance in the bone loss of PMO.^45^ Th17 cells are a subset of proinflammatory T‐helper cells that potently induce the differentiation of osteoclasts by releasing IL‐17A, RANKL, TNF and IL‐6,^46^, ^47^ consequently leading to bone destruction. Increased differentiation of Th17 cells and circulating IL‐17 levels have been observed in OVX mice and postmenopausal women.^4^, ^5^, ^6^ Neutralization of IL‐17A significantly prevents bone loss induced by oestrogen deficiency via coupling the bone‐remodelling process.^48^ On the other hand, oestrogen can promote the differentiation of Treg cells,^49^, ^50^ which is an immunosuppressive CD4^+^ T‐cell subsets mediating the tolerance to autoimmune and maintaining the immune homeostasis.^51^ Treg cells have been reported effectively inhibited the osteoclastogenesis via secreting TGF‐β, IL‐4 and IL‐10.^52^, ^53^ Treg cells can also induce the activation of enzyme indoleamine 2,3‐dioxygenase (IDO), initiating the apoptosis of osteoclast precursors via the cell‐cell contact mediated by cytolytic T lymphocyte‐associated antigen (CTLA‐4).^54^ The skewed distribution of Th17/Treg cells has been identified in the pathogenesis of PMO, as well as in other inflammatory diseases such as periodontal and periapical diseases. Function of Th17 and Treg is closely connected with bone metabolism.^46^, ^55^ Restoration of Th17/Treg balance has been proven effective in the management of CD4^+^ T cell‐mediated bone loss.^56^ The current study also identified skewed distribution of Th17/Treg cells as reflected by increased CD4^+^IL‐17A^+^ Th17 cells and decreased CD4^+^CD25^+^Foxp3^+^ Treg cells in the bone marrow of OVX rats, and intragastric administration of probiotics can effectively rescue this CD4^+^ T‐cell abnormality, consequently attenuating the alveolar bone loss as observed in either periodontitis or periapical periodontitis.

Recent studies have shown that the translocation of gut microbiota and/or its metabolites provides necessary antigens required for T‐cell activation in the bone marrow, inducing bone loss in oestrogen deficiency.^5^ The regulatory role of gut microbiota in osteoimmune has been recognized. In germ‐free mice, oestrogen deprivation failed to stimulate the osteoclastogenic cytokines and initiate the bone resorption.^5^ Components derived from gut microbiota, especially lipopolysaccharides (LPS), is a potent inflammatory inducer by activating Toll‐like receptors 4.^57^, ^58^ In addition, segmented filamentous bacteria (SFB), a group of spore‐forming gram‐positive microbes that colonize in the intestine of rodents including mice and rats, play a critical role in the induction of intestinal Th17 cells.^59^, ^60^, ^61^, ^62^ On the other hand, the short‐chain fatty acids (SCFAs) produced by gut microbiota not only represent as a major energy source for colonic epithelium, but also play an importantly role in the reinforcement of epithelial barrier functions via regulating TJ proteins.^63^, ^64^, ^65^ SCFAs also suppress the differentiation of Th17 cells and reduce the production of osteoclastogenic cytokines including IL‐6, IL‐17 and IL‐23.^66^, ^67^ In addition, SCFAs can induce the differentiation of colonic Treg cells via SCFA‐receptor FFAR2 (GPR43) and histone deacetylase (HDAC)‐inhibitory activity, thus suppressing the mucosal inflammation.^68^, ^69^ Promoting gut‐derived SCFAs generation has shown protective effects in multiple diseases including IBS, cardiovascular diseases, Alzheimer's disease and rheumatoid arthritis.^63^, ^70^, ^71^, ^72^ Probiotics have been shown to increase intestinal SCFAs levels and promote gut barrier function.^69^, ^73^, ^74^, ^75^ More importantly, probiotics have been reported to increase the bone mineral density.^24^ Probiotics can promote the absorption of bone formation‐related minerals,^76^, ^77^ suppress CD4^+^ T cells in bone marrow and protect bone loss induced by oestrogen deficiency.^78^ Intragastric administration of probiotics can also elevate Treg cells in the bone marrow of OVX mice and thus suppress bone loss.^79^, ^80^ Although mechanisms are still unclear, beneficial local effects of probiotics on inflammatory alveolar bone loss have also been reported.^35^, ^81^, ^82^, ^83^ Our previous study also showed that administration of berberine, a natural alkaloid with well‐known ecological effect on gut microbiota, can enrich butyrate‐generating bacteria and elevate the generation of gut‐derived SCFAs, which promote gut barrier function and attenuate OVX‐exacerbated periodontal bone loss.^38^ Consistently, our current work found that the dysbiosis of gut microbiota under oestrogen deficiency increased gut permeability with enhanced serum LPS, and the ensuing inflammatory responses skewed the distribution of Th17/Treg cells in the bone marrow and aggravated alveolar bone loss. Probiotics can reconstitute the structure of gut microbiota, particularly enrich butyrate‐generating bacteria and enhance the production of SCFAs, which promote gut barrier function and subsequently restore the Th17/Treg cell balance and consequently ameliorate alveolar bone loss both in either periodontal and periapical diseases. Moreover, administration of probiotics effectively decreased the level of SFB, which may further contribute to the alleviation of Th17‐associated alveolar bone resorption in the oestrogen‐deficient rats.

Of note, the current study showed that intragastric administration of probiotics exerted no positive effects on the alveolar bone destruction in rats free of OVX or inflammatory state. This is consistent with our previous findings of berberine or probiotics treatment outcome reported by others.^38^, ^84^ This may suggest the unnecessity of over‐supplementation of probiotics in healthy population as also suggested by many researchers.^85^, ^86^ In addition, the specific mechanisms by which probiotics enrich butyrate‐producing genera still need further investigation. To achieve better clinical outcome, more clinical studies are still needed to optimize the formula, dosage, and the way of administration of the probiotics. Besides, the regulatory effects of probiotics on gut‐derived oestrogens have been suggested, further suggesting its application to the combinatory management of osteoporosis and alveolar bone loss in postmenopausal women.^87^

Taken together, our current work shows that oestrogen deficiency increases gut permeability with microbiota dysbiosis and the ensuing systemic inflammatory response skews bone marrow Th17/Treg distribution and consequently aggravates alveolar bone loss in periodontal diseases and periapical diseases. Probiotics can reconstitute gut microbiota and promote gut barrier function, restore bone marrow Th17/Treg balance and consequently ameliorate alveolar bone loss. Probiotics may represent a promising adjuvant therapeutics to the combinatory management of PMO and periodontal/periapical bone loss in elder women.

## CONFLICT OF INTEREST

All authors state that they have no conflicts of interest.

## AUTHOR CONTRIBUTIONS

Xin Xu, Zhou Xuedong contributed to the study design and critically revised the manuscript. Leming Jia, Ye Tu and Xiaoyue Jia contributed to conception, data acquisition, analysis and interpretation and drafted the manuscript. Du Qian, Xin Zheng contributed to data analysis and critically revised the manuscript. Quan Yuan and Liwei Zheng critically revised the manuscript.

## ETHICAL APPROVAL

All animal procedures in this study were approved by the Ethics Committee of West China Hospital, Sichuan University (licence number WCHSIRB‐D‐2018‐150).

## Supporting information

Fig S1‐S4

## Data Availability

All data generated or analysed during this study are included in this article.
