# Supplementary material for: Probiotics ameliorate alveolar bone loss by regulating gut microbiota
Source: Cell Prolif. 2021 Jun 7;54(7):e13075. doi: 10.1111/cpr.13075 (PMC8249787; doi:10.1111/cpr.13075)
Supplement: Supplementary file 1 — Fig S1‐S4 [file CPR-54-e13075-s001.docx]

Probiotics Ameliorates Alveolar Bone Loss by Regulating Gut Microbiota

Leming Jia^1,2,#^, Ye Tu^1,2,#^, Xiaoyue Jia^1,3#^, Qian Du^1,2^, Xin Zheng^1,2^, Quan Yuan^1,4^, Liwei Zheng^1,3^, Xuedong Zhou^1,2,*^, Xin Xu^1,2,*^

1. State Key Laboratory of Oral Diseases & National Clinical Research Center for Oral Diseases, West China Hospital of Stomatology, Sichuan University, Chengdu, China

2. Department of Cariology and Endodontics, West China Hospital of Stomatology, Sichuan University, Chengdu, China

3. Department of Paediatric Dentistry, West China Hospital of Stomatology, Sichuan University, Chengdu, China

4. Department of Dental Implantology, West China Hospital of Stomatology, Sichuan University, Chengdu, China

#: co-first authors

*Corresponding Authors: Prof. Xuedong Zhou, State Key Laboratory of Oral Diseases, West China Hospital of Stomatology, Sichuan University, No. 14 Section 3, Renmin South Road, Chengdu, Sichuan 610041, China. Email: zhouxd@scu.edu.cn; Prof. Xin Xu, State Key Laboratory of Oral Diseases, West China Hospital of Stomatology, Sichuan University, No. 14 Section 3, Renmin South Road, Chengdu, Sichuan 610041, China. Email: xin.xu@scu.edu.cn.

**Supplemental figures and figure legends**


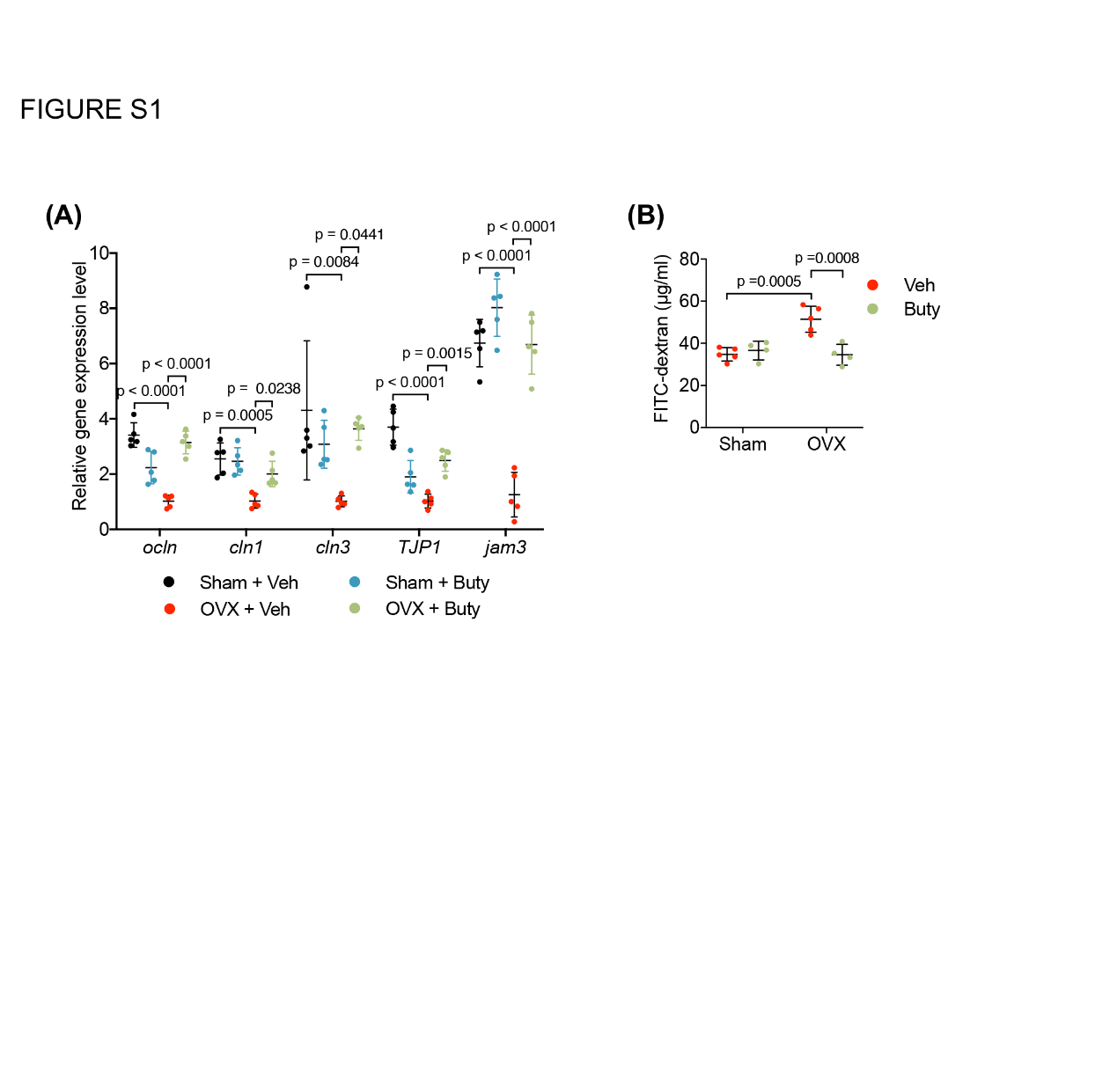


**FIGURE S1** Exogenous butyrate protects the gut permeability **(A)** The relative expression levels of intestinal epithelial TJ proteins as quantified by qRT-PCR. **(B)** Serum level of FITC-dextran. Data are presented as the mean ± SD, n = 5 rats per group. The p values are indicated above the horizontal bars. Buty, butyrate; TJ, tight junction; Veh, Vehicle.

**
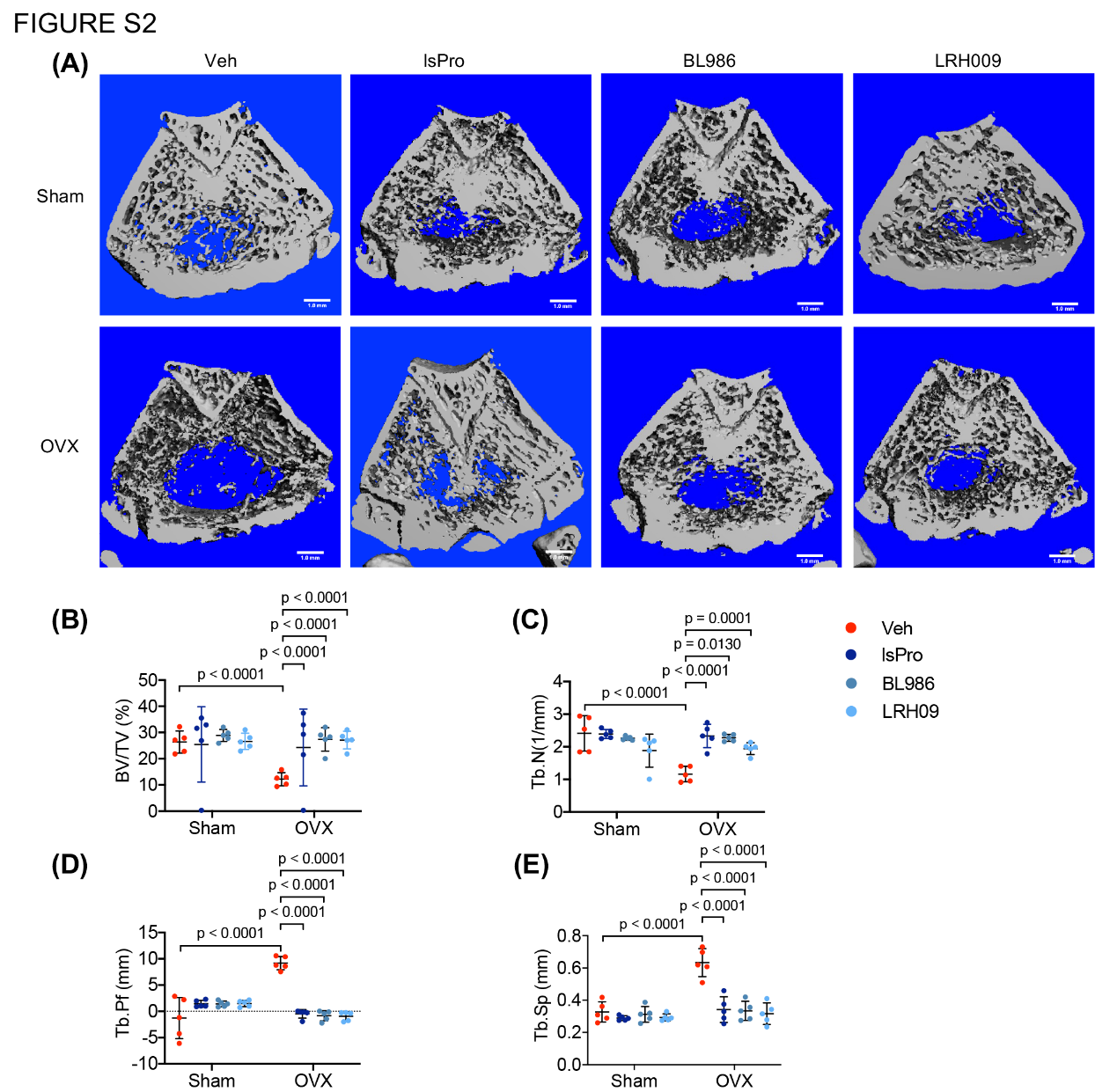
**

**FIGURE S2** Probiotics ameliorate femur bone loss in OVX rats. **(A)** Micro-CT reconstruction of femoral bone structure. **(B-E)** Quantitative analyses of BV/TV, Tb.N**,** Tb.Pf**,** Tb.Sp of the femur, respectively. Data are presented as the mean ± SD, n = 5 rats per group. The p values are indicated above the horizontal bars. BL986, *Bifidobacterium longum* BL986; BV/TV, bone volume per tissue volume; LRH09, *Lactobacillus rhamnosus* LRH09; lsPro, lifespace probiotics; Tb.N, trabecular number; Tb.pf, trabeculae pattern factor; Tb.Sp, trabecular separation.


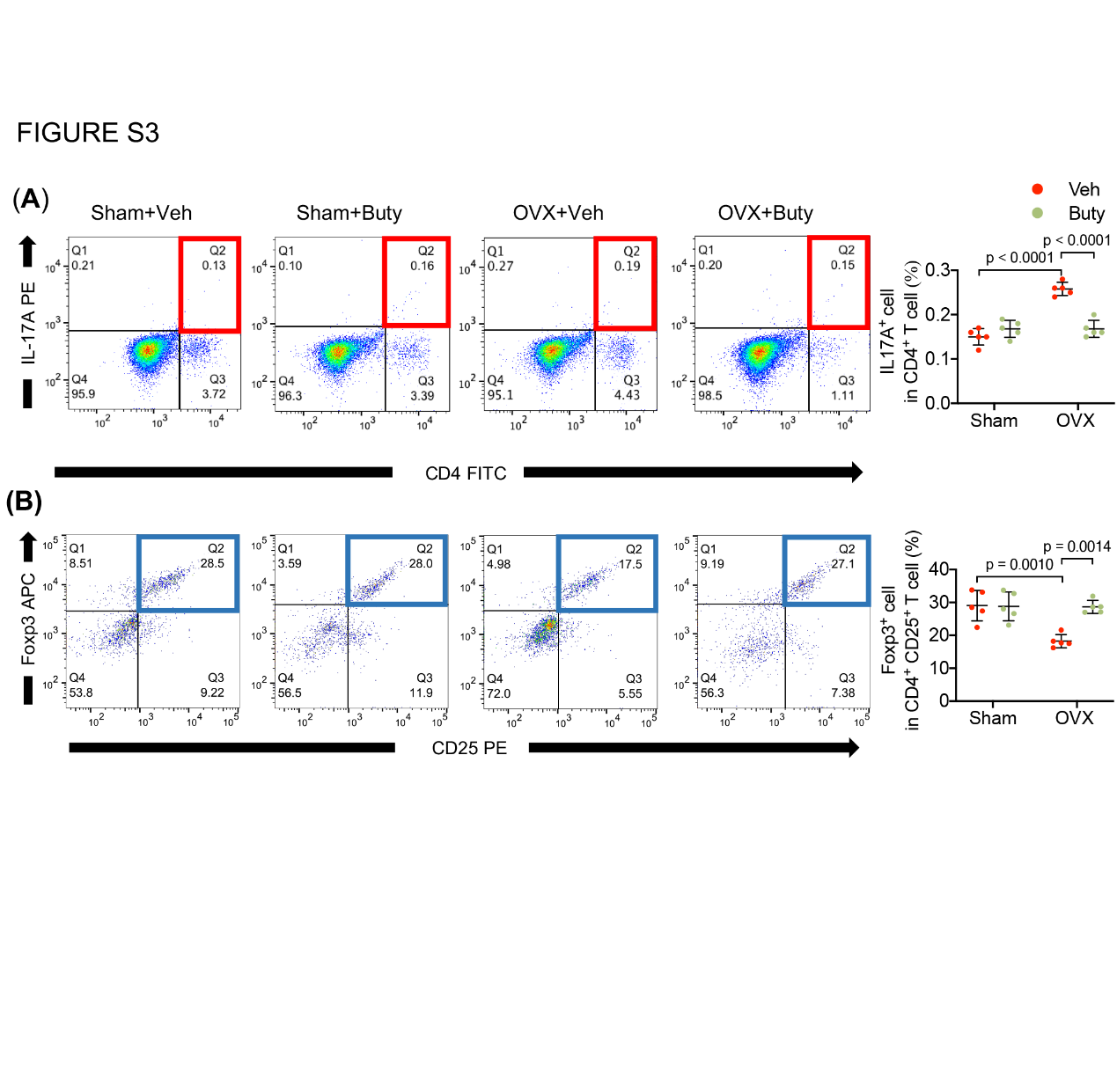


**FIGURE S3** Exogenous butyrate restores the distribution of Th17/Treg cells in bone marrow. **(A)** Representative FACS plots of IL-17A^+^ cells in the CD4^+^ T cell subset of the bone marrow, and quantitative analysis. **(B)** Representative FACS plots of the ratio of Foxp3^+^ cells in CD4^+^ CD 25^+^ T cell subset of the bone marrow and quantitative analysis. Data are presented as the mean ± SD, n = 5 rats per group. The p values are indicated above the horizontal bars. Buty, butyrate; Veh, Vehicle.


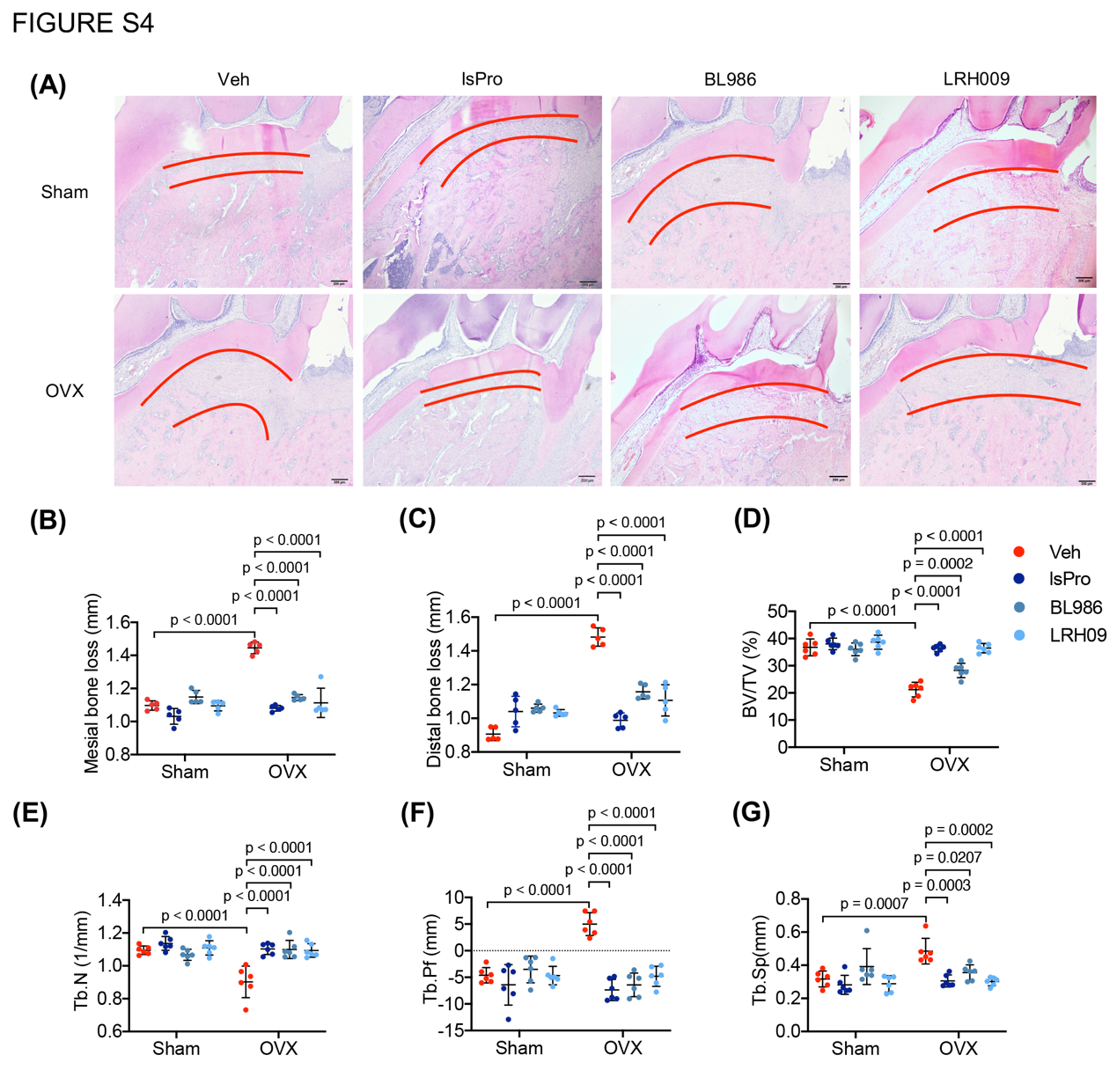


**FIGURE S4** Probiotics ameliorate periodontal bone loss in OVX rats. **(A)** H&E staining indicating alveolar bone loss at root furcation (distance between red lines). **(B, C)** Quantitative analyses of mesial and distal alveolar bone loss by micro-CT. **(D-G)** Micro-CT quantitative analyses of BV/TV, Tb.N, Tb.Pf, and Tb.Sp of the alveolar bone, respectively. Data are presented as the mean ± SD, n = 5 rats per group. The p values are indicated above the horizontal bars. BL986, *Bifidobacterium longum* BL986; BV/TV, bone volume per tissue volume; LRH09, *Lactobacillus rhamnosus* LRH09; lsPro, lifespace probiotics; Tb.N, trabecular number; Tb.pf, trabeculae pattern factor; Tb.Sp, trabecular separation.
